# Glucose and Serum Deprivation Led to Altered Proliferation, Differentiation Potential and AMPK Activation in Stem Cells from Human Deciduous Tooth

**DOI:** 10.3390/jpm12010018

**Published:** 2021-12-30

**Authors:** Madhura Pawar, Vivek Pawar, Apathsakayan Renugalakshmi, Ashraf Albrakati, Uthman S. Uthman, Harisha Dewan, Maryam Mugri, Mohammed Sayed, Shilpa Bhandi, Vikrant R. Patil, Rodolfo Reda, Luca Testarelli, Shankargouda Patil

**Affiliations:** 1 Department of Pediatric and Preventive Dentistry, Dr. D. Y. Patil Dental College and Hospital, Dr. D. Y. Patil Vidyapeeth, Pimpri, Pune 411018, India; drmadhura4@gmail.com; 2 Department of Oral & Maxillofacial Surgery, SMBT Institutes of Dental Sciences and Research, Dhamangaon, Nashik 422403, India; vivekpawar27@gmail.com; 3 Division of Pedodontics, Department of Preventive Dental Sciences, Jazan University, Jazan 45142, Saudi Arabia; arenu27@yahoo.co.in; 4 Department of Human Anatomy, College of Medicine, Taif University, Taif 21944, Saudi Arabia; a.albrakati@tu.edu.sa; 5 Department of Preventive Dental Sciences, College of Dentistry, Prince Sattam bin Abdulaziz University, Alkharj 11942, Saudi Arabia; Uthman.dental@yahoo.com; 6 Department of Prosthetic Dental Sciences, College of Dentistry, Jazan University, Jazan 45142, Saudi Arabia; harisha.dewan@yahoo.com (H.D.); drsayed203@gmail.com (M.S.); 7 Department of Maxillofacial Surgery and Diagnostic Sciences, College of Dentistry, Jazan University, Jazan 45142, Saudi Arabia; dr.mugri@gmail.com; 8 Department of Restorative Dental Sciences, College of Dentistry, Jazan University, Jazan 45142, Saudi Arabia; shilpa.bhandi@gmail.com; 9 Biogenre Private Limited, Pune 412105, India; patilvikrant.r@gmail.com; 10 Department of Oral and Maxillofacial Sciences, Sapienza University of Rome, 00185 Rome, Italy; rodolforeda17@gmail.com (R.R.); luca.testarelli@uniroma1.it (L.T.); 11 Department of Maxillofacial Surgery and Diagnostic Sciences, Division of Oral Pathology, College of Dentistry, Jazan University, Jazan 45142, Saudi Arabia

**Keywords:** nutrient deprivation, SHEDs, AMPK, quiescence, stemness

## Abstract

Stem cell therapy is an evolving treatment strategy in regenerative medicine. Recent studies report stem cells from human exfoliated deciduous teeth could complement the traditional mesenchymal stem cell sources. Stem cells from human exfoliated deciduous teeth exhibit mesenchymal characteristics with multilineage differentiation potential. Mesenchymal stem cells are widely investigated for cell therapy and disease modeling. Although many research are being conducted to address the challenges of mesenchymal stem cell therapy in clinics, most of the studies are still in infancy. Host cell microenvironment is one of the major factors affecting the homing of transplanted stem cell and understanding the factors affecting the fate of stem cells of prime important. In this study we aimed to understand the effects of serum deprivation in stem cells derived from human deciduous tooth. Our study aimed to understand the morphological, transcriptional, cell cycle and stemness based changes of stem cells in nutrient deprived medium. Our results suggest that stem cells in nutrient deprived media undergo low proliferation, high apoptosis and changed the differentiation potential of the stem cells. Serum deprived mesenchymal stem cells exhibited enhanced chondrogenic differentiation potential and reduced osteogenic differentiation potential. Moreover, the activation of key metabolic sensor AMP-activated kinase (AMPK) leads to activation of transcription factors such as FOXO3, which leads to an S phase quiescence. Serum deprivation also enhanced the expression of stemness related genes Sox2 and c-Myc.

## 1. Introduction

Stem cell therapy is one of the promising clinical approaches which will be a game changer for treating many diseases. However, there are so many challenges for the translating the technique from bench to bed side. Genetic instability of stem cells, immunological rejection by host immune system, stem cell culturing conditions, pharmacokinetic behavior of stem cells and ethical issues are some of the challenges in stem cell therapy [1]. Most of the stem cell therapies are still in preclinical trial stage and require extensive research for implementing in clinics. Obtaining viable and easily accessible stem cells is of prime important in stem cell research. Moreover, ethical issues associated with using embryonic stem cells (ESC) and human embryo also limit the research. Postnatal isolation of stem cells can be carried out from various tissues such as hair follicles, bone marrow, muscles and dental pulp [2,3,4]. Recently, Miura et al., showed human deciduous tooth as a source of multipotent stem cells and these stem cells from human exfoliated deciduous teeth (SHEDs) exhibited mesenchymal characteristics [5]. SHEDs are isolated from the dental pulp of physiologically shed deciduous tooth. As SHEDs are obtained without any invasive procedure, the molecular characteristics of SHEDs are widely under investigation for regenerative therapy [6]. Additionally, SHEDs possess high multipotency, proliferative capacity, minimum risk of oncogenesis and minimum ethical concerns [7,8].

Several studies have been carried out for characterizing the molecular features of SHEDs. Characterization of SHEDs will enable to understand the molecular properties as well as clinical potential of SHEDs. The proliferation and multipotential differentiation capacity of SHEDs have been shown in in-vitro and in-vivo models. SHEDs can differentiate into multiple lineages such as adipocytes, osteoblasts, endothelial cells, and neuronal-like cells [9,10]. The potential of SHEDs in repairing damaged tooth, induction of bone regeneration and to treat neural tissue injuries as well as degenerative diseases are under study [11]. It has been reported that SHEDs have higher proliferative aptitude than bone marrow derived mesenchymal stromal cells. SHEDs also possess high osteogenic differentiation potential than the stem cells from human dental pulp [12]. Gene expression profile of SHEDs showed high expression of genes associated with high proliferation [12]. Mesenchymal characteristics of SHEDs were also investigated and proposed SHEDs as an alternative source of Mesenchymal stem cells (MSCs). MSCs are multipotent stem cells which can be for tissue repair and for many clinical applications including cancer treatment. The Currently, several clinical trials have been carried out for demonstrating the efficacy of MSC in clinical application [13]. In different preclinical trials MSCs show their potential in treating bone and cartilage diseases, cancer, liver diseases and autoimmune disorders [14]. MSC based therapies for treating severe COVID-19 patients are also being under clinical trial [15].

There are many factors which affect the differentiation potential of the MSC. Heterogeneity of MSCs, variation in expansion capacity of MSCs due to different culture conditions, immune status of the recipient is some of them [15]. The microenvironment of MSCs affect the differentiation fate of MSCs [15]. Additionally, MSCs are highly glycolytic and glucose deprivation and nutrient supply due to damaged blood vessels during tissue regeneration is one of the major challenges [16,17]. Therefore, understanding the effect of low glucose and nutrients on MSCs will be immensely useful. It has been reported that MSCs, increased reactive oxygen species (ROS) can reduce the proliferative potential, increase senescence, enhance adipogenic but reduce osteogenic differentiation [9]. A study conducted on human bone marrow-derived MSCs showed that serum mediated oxidative stress can affect the function of the mesenchymal cells [10]. Serum deprivation has many effects in cells, for example in Balb/c 3T3 fibroblasts cells serum deprivation led to induction of apoptosis [18]. However, the effect of serum deprivation and low glucose on SHEDs is not yet explored.

In this study, we aimed to study the effect of low glucose and serum deprivation on MSCs derived from SHEDs. We isolated and characterized SHEDs and further checked the multilineage differentiation potential of the isolated SHEDs. Low glucose and serum treatment resulted in decreased proliferation, alteration in differentiation potential and increased apoptosis in SHEDs. Further, we observed activation of AMPK, upregulation of FOXO3 upregulation and cell cycle arrest in S-phase. Our results suggest, the serum deprivation and low glucose affect the fate of SHEDs in in-vitro models.

## 2. Materials and Methods

### 2.1. Sample Collection

Human exfoliated deciduous teeth were retrieved from healthy 6–12 years old subjects who practiced good oral care. The institutional ethical committee of Dr. D. Y. Patil Dental College and Hospital approved the study (Ref. No.: DYPDCH/41/2018), and written consent was acquired.

### 2.2. Culture and Expansion of Stem Cells from Human Exfoliated Deciduous Teeth (SHEDs)

Cell culture refers to the removal of cells from an animal or plant and their subsequent growth in a favorable artificial environment. The cells may be removed from the tissue directly and disaggregated by enzymatic or mechanical means before cultivation, or they may be derived from a cell line or cell strain that has already been already established. Primary culture refers to the stage of the culture after the cells are isolated from the tissue and proliferated under the appropriate conditions until they occupy all of the available substrate. The explant culture methodology, which was previously described by Patil et al., 2018, was explored for the isolation and characterization of SHEDs. The tissue was fragmented and fetal Bovine serum (FBS) (Gibco, Rockville, MD, USA) was supplemented to cover the pieces. The cells are then incubated for 24 h at 37 °C with 5% CO_2_ and then kept in DMEM (Invitrogen, Carlsbad, CA, USA) (20 percent FBS and antibiotic and antimycotic supplements). The culture medium was changed two times per week, and an inverted phase-contrast microscopy was utilized to examine cell health, morphology, and growth on a regular basis. Subculturing was done at 70–80% confluence using 0.25 percent trypsin-EDTA solution (Invitrogen, Carlsbad, CA, USA). The cells were then transplanted to a larger polystyrene culture flask with a surface area of 25 cm2 (Nunc, Rochester, NY, USA). Complete growth medium (DMEM + 10% FBS) was used to keep the SHEDs growing. In the experimental procedures, cells from passages 2 to 4 were used.

### 2.3. Experimental Groups

For control group, SHEDs were cultured in DMEM + 10% FBS and For LowGS group, SHEDs were cultured in DMEM (no glucose) + 0.2 g/L glucose + 2% FBS. All the experiments were carried out for day 15 time point except cell count was carried out each single day for 13-day time points. The media was changed with fresh medium every day in all experimental groups.

### 2.4. Cell Surface Marker Analysis of SHEDs by Flow Cytometry

Flow cytometry is a biophysical technology used in cell counting, sorting and biomarker detection by measuring optical and fluorescence characteristics of single cells. Trypsinization was used to collect confluent SHEDs, which were then rinsed twice with PBS. Anti-human-CD29-PE, anti-human-CD90-APC, anti-human-CD73-APC, anti-human-CD105-APC, anti-human-CD140b-PE, anti-human-CD271-FITC, anti-human-CD34-PE, anti-human-CD45-FITC, anti-human-STRO-1-APC, and anti-human-HLA-DR-APC antibodies (Miltenyi Biotec, Bergisch Gladbach, Germany) were then incubated for 30 min at 4 °C. Antibody-labelled cells were given a wash twice in PBS beforehand being acquired on the low cytometry system at a rate of 10,000 cells per sample (Attune, Thermo Fisher Scientific, Waltham, MA, USA). To identify and distinguish between negative or positive signals, isotype controls were used.

### 2.5. Osteogenic Differentiation

Multipotent mesenchymal stem cells show differentiation potential into multiple lineage cells especially osteoblasts, adipocytes and chondrocytes. A 24-well cell culture plate (Nunc, Rochester, NY, USA) along with complete growing medium was employed through a cell density of 2500 cells/cm^2^. The media was replaced with DMEM containing 1% antibiotic-antimycotic, 10 mM β-glycerophosphate, 50 µM ascorbate-2-phosphate, and 0.1 µM dexamethasone after 24 h (Sigma-Aldrich Corp., St. Louis, MO, USA). The medium is refreshed twice a week with induction medium. The cells were fixed with 4 percent paraformaldehyde and stained with 2 percent alizarin red S (pH 4.1–4.3) for 20 min to observe differentiation to osteogenic lineage after 21 days.

### 2.6. Differentiation into Adipocytes

SHEDs were plated on a 24-well cell culture plate (2500 cell per cm^2^ surface area) (Nunc, Thermo Fisher, Rochester, NY, USA) using complete growing medium. Subsequently 24 h after the seeding, the cells were given adipogenic medium (DMEM supplemented with 10% FBS, 0.5 mM isobutyl-methylxanthine, 10 µM insulin, 1 µM dexamethasone, 200 µM indomethacin, and (Sigma-Aldrich Corp., St. Louis, MO, USA) twice weekly for three weeks. Differentiated cells into adipocytes were fixed by using 4% paraformaldehyde for 1 h and then verified with 0.3 percent oil red O to reveal lipid condensations produced in the cells.

### 2.7. Chondrogenic Differentiation

A 24-well cell culture plate (Nunc, Rochester, NY, USA) along with complete growing medium was employed with a cell density of 2500 cells/cm^2^. After 24 h, the entire growth media was replaced with DMEM containing 1 mM sodium pyruvate, 1 × -ITS, 100 nM dexamethasone, 40 µg/mL L-proline, 10 ng/mL TGF-β3, and 50 µg/mL ascorbate-2-phosphate (Sigma-Aldrich Corp., St. Louis, MO, USA). The cultures were cultured for 28 days at 37 °C in a 5% CO_2_ incubator, with the media being replenished every 2–3 days. After fixing the cells with 4 percent paraformaldehyde, the cells were exposed to 0.1 percent safranin O for 30 min.

### 2.8. Growth Curve Plotting

Mesenchymal stem cells show rapid proliferation potential. Growth curve plotting can help in determining population doubling time. 1 × 10^4^ passage 2 cells were introduced onto 12-well plates and the counting of cells was performed every other day for 15 days of incubation to check proliferative ability of SHEDs. Cell counts were recorded for 15 days and the growth curve was displayed.

### 2.9. Analysis of Cell Cycle by Flow Cytometry Method

Cellular proliferation can be assessed by measuring the different stages of the cell cycle in a population of cells. The DNA content varies with each phase of the cell cycle and this can be assessed using fluorescent DNA binding dyes and monoclonal antibodies to detect the expression of antigens. Flow cytometry was used to assess the distribution of cell cycle phases in the cells on the basis of DNA content. SHEDs were plated at 5 × 10^4^ cells per well in 12-well plates. After that, the cells were rinsed with PBS and fixation was done by using 70% ice cold ethanol for 2 h at 4 °C. The fixed cells were given a treatment of RNase A (at 10 mg/mL concentration) and kept in the dark for 30 min at room temperature with propidium iodide (PI) treatment. Flow cytometry was used to quantify the PI fluorescence of individual nuclei, and the cell percentage in different phases of the cell cycle was estimated.

### 2.10. Quantitative Analysis of Expression of Genes by Real Time Quantitative Polymerase Chain Reaction (RT-qPCR)

RT-qPCR is a method used to amplify cDNA copies of RNA. Sensitive and versatile, RT-qPCR is used to retrieve and clone the 5’ and 3′ termini of mRNAs and to generate large cDNA libraries from very small amounts of mRNA. In addition, RT-PCR can be easily adapted to identify mutations and polymorphisms in transcribed sequences and to measure the strength of gene expression when the amounts of available mRNA are limited and/or when the RNA of interest is expressed at very low levels. The total RNA of the cells was purified using the GeneJET RNA purification kit for RT-qPCR (Thermo Scientific, Vilnius, Lithuania). RNA (2 µg) was reversely transcribed according to the instructions by manufacturer with a cDNA synthesis kit (High Capacity, Applied Biosystems, Carlsbad, CA, USA). For individual gene of interest, 100 ng cDNA was employed in a complete reaction volume of 20 µg. With a Real-Time PCR system (Quant Studio 5, Applied Biosystems, Foster City, CA, USA), quantitative examination of genes of interest was worked out by utilizing SYBR Green universal PCR master mix reagent (Applied Biosystems, Thermo Fisher, Austin, TX, USA). The ΔΔCt calculation was used to normalize target gene expression to ACTB as a reference gene. Table 1 contains a list of genes as well as primers (Eurofins).

### 2.11. Mitochondrial Potential Analysis by Flow Cytometry Method

Flow cytometry can be used to evaluate changes that occur in cells over short periods of time. Biological activities such as change in calcium content in response to drugs; the generation of reactive oxygen species or mitochondrial membrane changes during apoptosis and phagocytosis rates using labelled bacteria, can all be assessed. To determine mitochondrial membrane potential in the cells, the MitoProbe DiIC1(5) kit (Thermo Fisher Scientific, Waltham, MA, USA) was utilised. In eukaryotic mitochondria, DiIC1(5) accumulates in relation to mitochondrial membrane potential. The cells were plated at a density of 1 × 10^6^ cells in each well in 12-well plates and treated with complete media for 24 h. The cells were treated with 10 µM of 1,1′,3,3,3′,3′-hexamethylindodicarbo-cyanine iodide (DiIC1(5)) after incubation. Cells for negative control were cultured at 37 °C with 5% CO_2_ for 15–30 min after being treated with 50 µM Carbonyl cyanide 3-chlorophenylhydrazone (CCCP) (Thermo Fisher Scientific, Waltham, MA, USA), a protonophore mitochondrial toxin. The cells were given a wash with PBS after incubation before being examined with a flow cytometer. The median fluorescence intensity was used to calculate the data (MFI).

### 2.12. Analysis of Cell Apoptosis Using Annexin V-FITC/PI Assay by Flow Cytometry Method

Flow cytometry can be used to detect the characteristic morphological and biochemical changes that occur during apoptosis. The apoptotic assay was carried out using Annexin V-FITC/PI labelling (BD Pharmingen, San Diego, CA, USA). The cells were plated at a density of 1 × 10^5^ cells per well in 12-well plates and incubated for 24 h. The cells were collected and stained with Annexin V-FITC reagent, both treated and untreated. In the absence of light, the cells were incubated for 15 min at room temperature. After incubation, the cells were exposed to PI reagent and flow cytometry (Attune NxT, Thermo Fisher Scientific, Waltham, MA, USA) was used to determine the percentage of apoptotic cells.

### 2.13. Western Blot Analysis

Western blot technique is used to transfer fragments of proteins from an electrophoresis gel to a membrane. It helps in detecting the proteins of interest expressed by the cells. Cells were lysed and the pellet centrifuged at 12,000 rpm for 15 min for western blot analysis. In the supernatant, protein was estimated, and an equal amount of protein was measured and placed onto the gel. AMPK, p-AMPK, and GAPDH antibodies (Abcam, Cambridge, UK) were employed as primary antibodies, and HRP-conjugated secondary antibodies were utilised to detect proteins of interest AMPK, p-AMPK, and GAPDH using the Western blot method.

### 2.14. Statistical Analysis

For all samples, the data were provided as the mean and standard deviation of three separate experimental values. For each cytokine, the data were evaluated with the help of two-tailed t test (unpaired) on the Prism software v8 (GraphPad Software, La Jolla, CA, USA), with *p* < 0.05 being designated significant and *p* < 0.01 being highly significant (ns not significant, * *p* < 0.05, and ** *p* < 0.01).

## 3. Results

### 3.1. SHEDs Show MSC-like Morphology, Cell Surface Marker Expression, and Trilineage Differentiation

**Figure 1:**
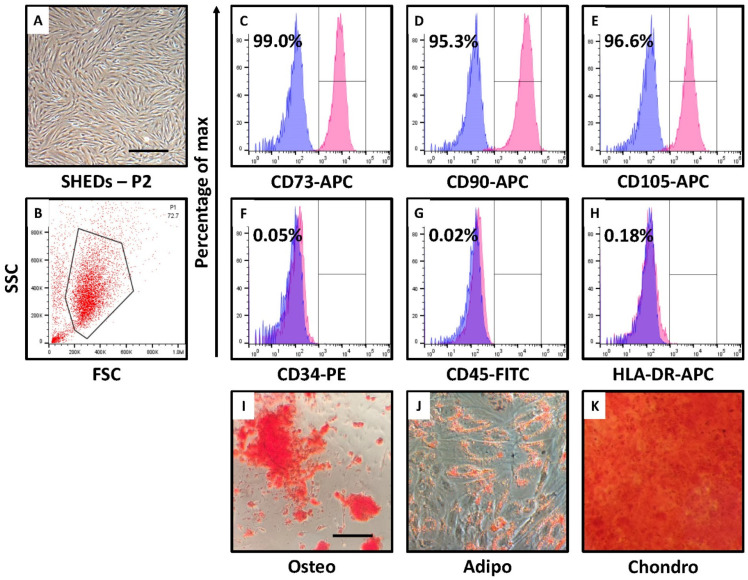
Isolation, characterization flow cytometry for MSCs associated cell surface markers, and tri-lineage differentiation of SHEDs. (**A**) Photomicrograph of passage 2 SHEDs. Scale bar = 100 μm. (**B**–**H**) Characterization of SHEDs for MSC-specific positive cell surface markers CD73, CD90, CD105 and negative markers CD34, CD45, CD105. (**I**–**K**) Differentiation of SHEDs into osteoblasts, adipogenic lineage, and chondrogenic lineage. Scale bar = 100 µm. SHEDs: Stem cells from exfoliated deciduous tooth, Osteo: Osteogenic induction, Adipo: Adipogenic induction, Chondro: Chondrogenic induction.

The SHEDs derived using the explant culture method is further characterized for the stemness as well as mesenchymal properties. The morphological analysis of SHEDs showed MSC-like characters under microscope (Figure 1A), positive marker expression of MSC-specific cell surface markers. We analyzed the expression of MSC markers such as CD73, CD90, and CD105, and we found these cells presented positive marker expression for the MSC markers (Figure 1B–E), whereas non-MSC markers CD34, CD45 and MHC Class II antigen and HLA-DR showed negligible marker expression in SHEDs (Figure 1F–H). Furthermore, we checked the differentiation potential of the derived SHEDs. We could observe successful differentiation of SHEDs into adipocytes, chondrocytes, and osteoblasts, which validates the effective isolation of SHEDs (Figure 1I–K).

### 3.2. Expression of CD29, STRO-1, CD271 and CD140b in Nutrient Deprived SHEDs

**Figure 2:**
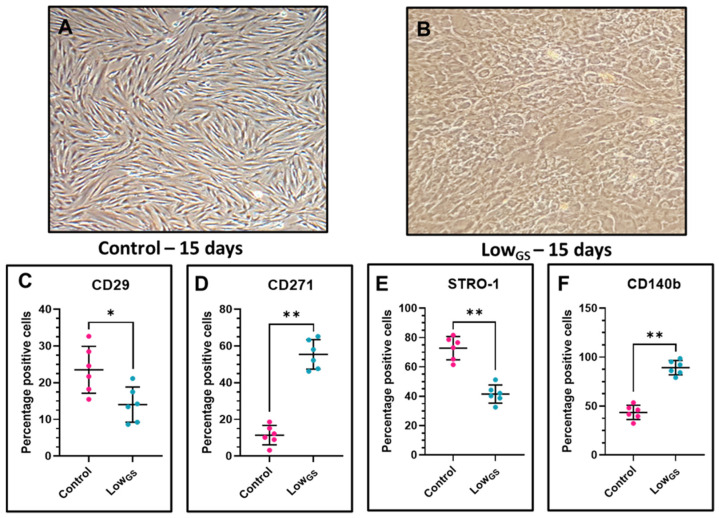
(**A**,**B**) Morphological characteristics and cell surface marker analysis by flow cytometry of SHEDs. (**C**–**F**) Cell surface marker analysis of SHEDs incubated with complete growth medium (Control) and very low glucose-serum medium (LowGS) for 15 days. * *p* < 0.05, ** *p* < 0.01. Control: SHEDs incubated with complete growth medium (DMEM + 10% FBS), LowGS: SHEDs incubated with very low glucose-serum medium (DMEM-no glucose + 0.2 g/L glucose + 2% FBS).

Further, we intended to check the effects of nutrient deprivation and low glucose level in SHEDs. The incubation of SHEDs with low glucose serum media leads to the change in morphological characteristics of the SHED cells compared with the control cells (Figure 2A,B). We analyzed the expression of epithelial marker CD29, mesenchymal and osteogenic differentiation marker STRO1, bone marrow MSC marker CD271 and mesenchymal marker CD140b (Figure 2C–F). We observed low expression of CD29 and STRO-1 in SHEDs incubated with low glucose serum medium but increased expression of CD271 and CD140b than SHEDs incubated with complete growth medium.

### 3.3. Serum Deprivation Induced Cell Cycle Arrest with Cell Cycle Synchronization in S Phase

**Figure 3:**
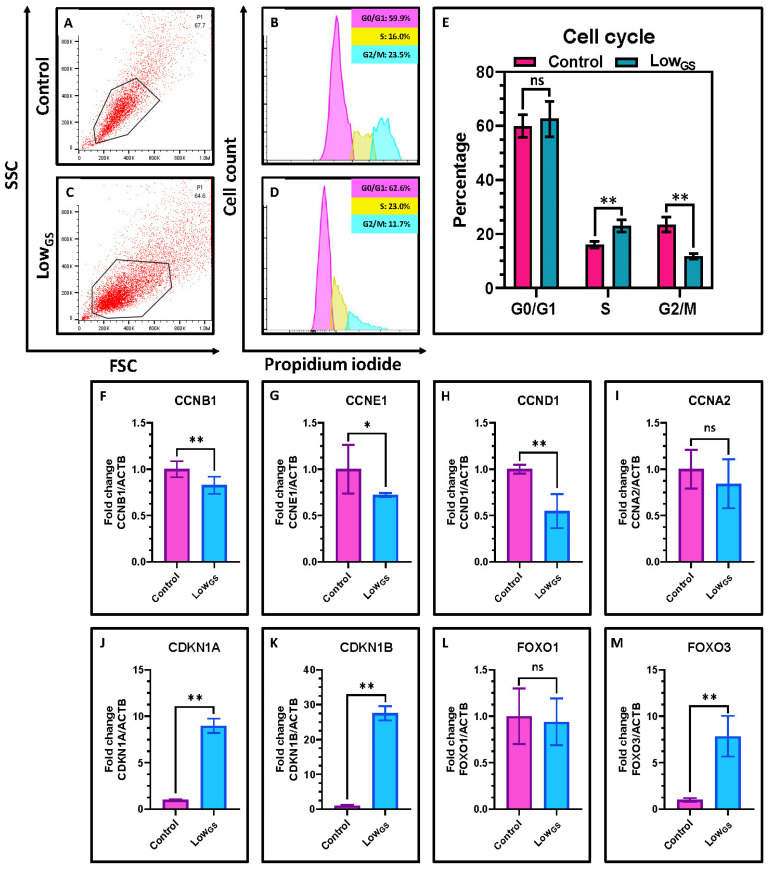
Cell cycle analysis by flow cytometry and analysis of gene expression by quantitative RT-qPCR in SHEDs. (**A**–**E**) Comparative cell cycle analysis of SHEDs incubated with complete growth medium (Control) and very low glucose-serum medium (LowGS) for 15 days. (**F**–**M**) Comparative gene expression analyses for cell cycle and quiescence-related genes CCNB1, CCNE1, CCND1, CCNA2, CDKN1A, CDKN1B, FOXO1, and FOXO3 in SHEDs incubated with complete growth medium (Control) and very low glucose-serum medium (LowGS) for 15 days. ns not significant, * *p* < 0.05, ** *p* < 0.01. Control: SHEDs incubated with complete growth medium (DMEM + 10% FBS), LowGS: SHEDs incubated with very low glucose-serum medium (DMEM-no glucose + 0.2 g/L glucose + 2% FBS), CCNB1: G2/mitotic-specific cyclin-B1, CCNE1: G1/S-specific cyclin-E1, CCND1: G1/S-specific cyclin-D1, CCNA2: Cyclin-A2, CDKN1A: Cyclin-dependent kinase inhibitor 1A, CDKN1B: Cyclin-dependent kinase inhibitor 1B, FOXO1: Forkhead box protein O1, FOXO3: Forkhead box protein O3.

We further investigated the effect of serum starvation in cell cycle. The flow cytometric analysis revealed most the serum deprivation retained a large portion of cells in S-phase compared to the control (Figure 3A–E). We also observed a decrease in the percentage of cells in G2/M phase in serum deprived condition. Whereas, there was no significant difference in number of cells in G0/G1 between the both conditions. Additionally, we studied the expression of cyclins, cell cycle inhibitors and proteins involved in cell cycle in these SHEDs. We observed low expression of cyclins such as cyclin B1 (CCND1), cyclin E1(CCNE1) and cyclin B1 (CCNB1) (Figure 3F–H). But there was no significant difference between cyclin A2 (CCNA2) between the two groups (Figure 3I). We further assessed the expression cyclin dependent kinase inhibitors CDKN1A and CDKN1B. We observed high expression of both the kinase inhibitors in the nutrient deprived SHEDS (Figure 3J,K). Further, we observed high expression of transcription factor FOXO3 in the nutrient deprived SHEDs, however there is no difference in expression of FOXO1 (Figure 3L,M).

### 3.4. Serum Deprived SHEDs Exhibited Decreased Proliferation, Mitochondrial Activity and Increased Apoptosis

**Figure 4:**
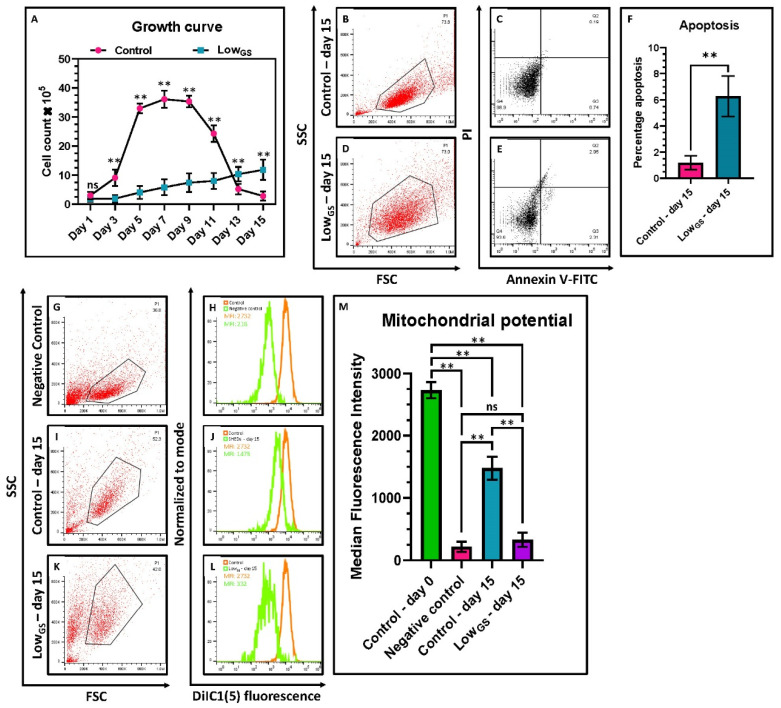
Cell proliferation by cell counting, apoptosis and mitochondrial potential by flow cytometry in SHEDs. (**A**) Comparative growth curves of SHEDs incubated with complete growth medium (Control) and very low glucose-serum medium (LowGS) for 13 days. (**B**–**F**) Comparative percentage apoptosis in SHEDs incubated with complete growth medium (Control) and very low glucose-serum medium (LowGS) for 15 days. (**G**–**M**) Comparative mitochondrial potential (MFI) in SHEDs incubated with complete growth medium (Control) and very low glucose-serum medium (LowGS) for 15 days. Carbonyl cyanide 3-chlorophenylhydrazone (CCCP) was used as negative control. ns not significant, ** *p* < 0.01. Control: SHEDs incubated with complete growth medium (DMEM + 10% FBS), LowGS: SHEDs incubated with very low glucose-serum medium (DMEM-no glucose + 0.2 g/L glucose + 2% FBS).

We analyzed the cell proliferation rate between the serum deprived SHEDs compared to the control (Figure 4A). We observed serum deprived SHEDs exhibited decreased proliferation. SHED in nutrient deprived condition exhibited low mitochondrial activity (Figure 4G–M). We further the effect of serum deprivation on apoptosis, we found that the low-glucose serum medium enhances the apoptosis in SHEDs (Figure 4B–F).

### 3.5. Nutrient Deprivation Leads to the Activation of AMPK in SHEDs

**Figure 5:**
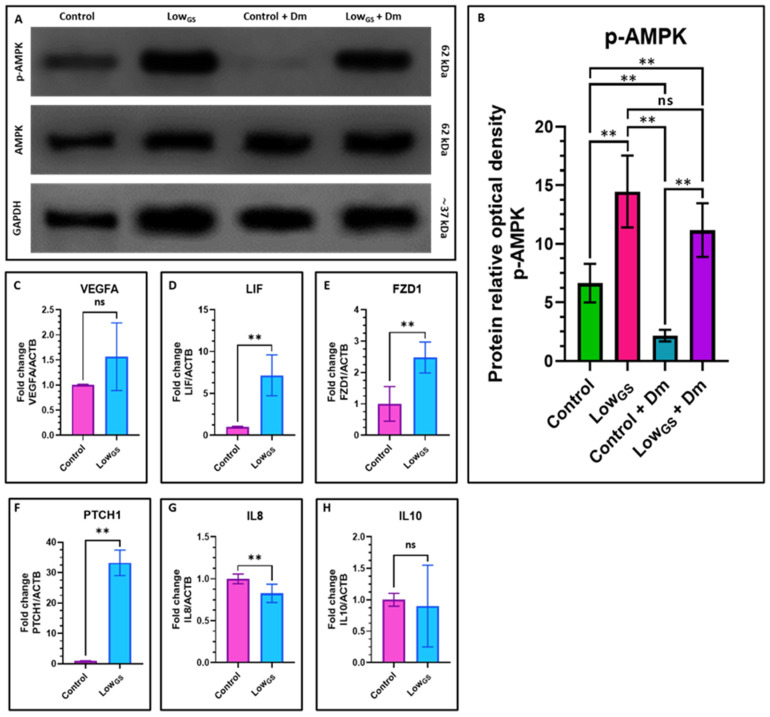
Protein expression by the Western blot and gene expression analysis by quantitative RT-qPCR in SHEDs. (**A**,**B**) Comparative protein expression (relative protein signal intensity) for AMPK and phosphorylated AMPK in in SHEDs incubated with complete growth medium (Control), very low glucose-serum medium (LowGS), and both the groups treated with dorsomorphin (selective AMPK inhibitor) for 15 days. (**C**–**H**) Comparative gene expression analyses for trophic factors, cytokines, and pluripotency-related genes VEGFA, LIF, IL8, IL10, FZD1, and PTCH1 in SHEDs incubated with complete growth medium (Control) and very low glucose-serum medium (LowGS) for 15 days. ns not significant, ** *p* < 0.01. Control: SHEDs incubated with complete growth medium (DMEM + 10% FBS), LowGS: SHEDs incubated with very low glucose-serum medium (DMEM-no glucose + 0.2 g/L glucose + 2% FBS), Dm: Dorsomorphin (Compound C), VEGFA: Vascular endothelial growth factor A, LIF: Leukemia inhibitory factor, IL8: Interleukin 8/C-X-C motif chemokine ligand 8, IL10: Interleukin 10/cytokine synthesis inhibitory factor, FZD1: Frizzled class receptor 1, PTCH1: Protein patched homolog 1.

Glucose starvation activates 5′ AMP-activated protein kinase (AMPK) which is essential for plays a critical role in maintaining redox homeostasis. So, we further aimed to check the expression of AMPK in nutrient deprived SHEDs. The gene and protein analysis of AMPK revealed elevated expression of AMPK and phosphorylated AMPK respectively (Figure 5A,B).

Further, the gene expression analysis of trophic factors, cytokines, and pluripotency-related genes Vascular endothelial growth factor A (VEGFA), Leukemia inhibitory factor (LIF), Interleukin 8/C-X-C motif chemokine ligand 8 (IL8), Interleukin 10/cytokine synthesis inhibitory factor (IL10), Frizzled class receptor 1 (FZD1), Protein patched homolog 1 (PTCH1). We observed high expression of LIF, FZD1 and PTCH1 in SHEDs incubated with low glucose (Figure 5D–F). However, the we observed low expression of expression of IL8 in the nutrient and glucose deprived cells. (Figure 5G). There was no significant difference in expression between VEGFA and IL10 between the two groups (Figure 5C,H).

### 3.6. Nutrient Deprivation Elevated the Expression of Stemness Genes and Chondrogenic Differentiation in SHEDs

**Figure 6:**
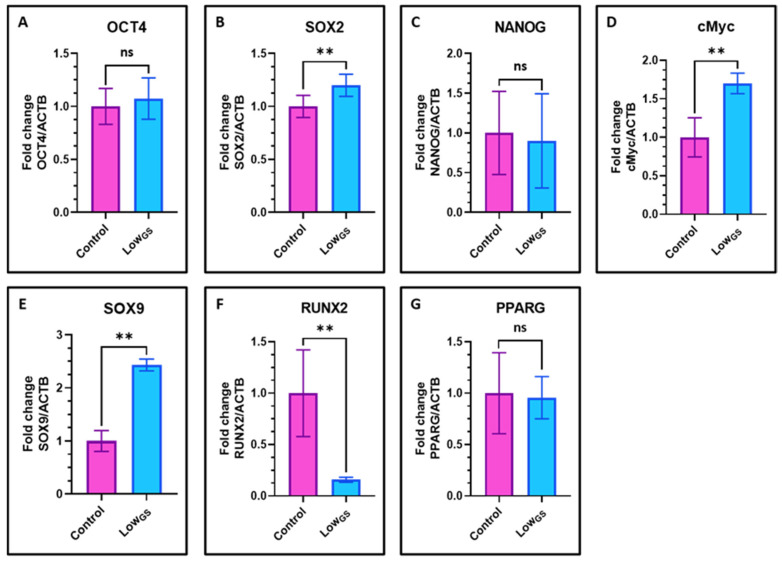
Gene expression analysis by quantitative RT-qPCR in SHEDs. (**A**–**G**) Comparative gene expression analyses for stemness and differentiation-related genes OCT4, SOX2, NANOG, MYC, SOX9, RUNX2, and PPARG in SHEDs incubated with complete growth medium (Control) and very low glucose-serum medium (Low_GS_) for 15 days. ns not significant, ** *p* < 0.01. Control: SHEDs incubated with complete growth medium (DMEM + 10% FBS), Low_GS_: SHEDs incubated with very low glucose-serum medium (DMEM-no glucose + 0.2 g/L glucose + 2% FBS), OCT4: Octamer-binding transcription factor 4, SOX2: SRY (sex determining region Y)-box 2, NANOG: Homeobox protein NANOG, MYC: MYC proto-oncogene/BHLH transcription factor, SOX9: SRY (sex determining region Y)-box 9, RUNX2: Runt-related transcription factor 2, PPARG: Peroxisome proliferator- activated receptor gamma.

To evaluate the stemness potential of the nutrient deprived SHEDs we examined the expression of gene expression of stemness related genes. SHEDs incubated with very low glucose-serum medium demonstrate upregulated expression of stemness associated genes including Sox2 and c-Myc but not Oct4 and Nanog (Figure 6A–D). We further checked the expression of regulators chondrogenesis and osteogenesis in SHEDs. We observed high expression of Sox9, which is master regulator of chondrogenesis (Figure 6E) and downregulation of osteogenesis regulator RUNX2 (Figure 6F).

## 4. Discussion

In regenerative medicine, cell therapy is regarded as a promising strategy to treat the diseases which are caused by cell death. In recent years, the development of treatment methods has been a great hope. However, currently, there are many challenges for implementing stem cell-based therapy widely. One of the most important challenge is the complete functioning and fate of stem cells in the transplanted system. There are many factors which determine the fate of stem cells and the prime important one is the microenvironment. Recently it has been shown that, the tissue microenvironment can determine the proliferation, cell survival, and even de-differentiation of transplanted stem cells [19].

Mesenchymal stem cells (MSCs)due to their ability to differentiate into cells of different mesenchymal origin, it is widely under investigation in cell therapy. SHEDs obtained from the pulp of human deciduous pulp open a new avenue, due to the effortlessness of getting the samples and less ethical issues. The multilineage differentiation potential and proliferation potential of SHEDs compared to bone marrow MSCs suggests SHEDs can complement bone marrow MSCs [20]. The success of MSC based cell therapy primarily depends on factors such as status of host immune status, the microenvironment. The microenvironment factors such as inflammation, hypoxia, and extracellular matrix influence the homing and fate of transplanted MSCs [15]. In present study, we aimed to identify the role of serum deprivation and low glucose in the determining the differentiation potential of MSCs.

The isolation of the SHEDs was carried out from the human deciduous teeth pulp and characterized. The morphological as well as cell surface marker-based characterization of SHEDs revealed mesenchymal property with a potential to differentiate into adipogenic, osteogenic and chondrogenic cell lineages. Further to comprehend the effect of nutrient deprivation and low glucose levels in SHEDs we incubated the cells in same conditions. The incubation of SHEDs in nutrient deprived and low glucose medium leads to change in morphology of the cells. Although, phenotypically the cells were not showing mesenchymal morphology these cells showed, low expression of epithelial marker CD29, high expression of mesenchymal markers CD271 and pluripotent stem cell marker CD140b. These indicates the changes in cell morphology may be an adaptation to survive the low nutrient medium. Changes in cell morphology associated with low nutrients is previously reported in primary microglia and BV-2 cells [21]. CD140b is also an angiogenic mediator which might be activated due to the low glucose level [22]. It has been shown hypoxia and serum deprivation can induce angiogenesis [23]. Serum deprivation and low glucose level also reduced the differentiation potential of MSCs to osteogenic lineage. Our results suggest the combined effect of nutrient deprivation and glucose can affect the fate and multipotency of SHEDs.

Previous studies have shown that nutrient deprivation leads to the cell cycle synchronization [24]. So, we aimed to study the effects of external factors in regulation of cell cycle phases in SHEDs. Our results showed most of the cells are arrested in the S-phase of the cycle. Tinnemans et al., previously reported S-phase cell cycle arrest in lung cancer cells due to nutrient deprivation [25]. Further we observed low expression mitosis specific cyclin, cyclin B; Cyclin E (G1 phase progression and S phase entry); cyclin D (G1/S phase transition cyclins) in these cells. We witnessed high expression of kinase inhibitors such as CDKN1A (p21) and CDKN1B (p27). High expression of p21 is related to cell cycle arrest or quiescence. P21 inhibit the activity of cyclin dependent kinases and arrest the cell cycle progression [26]. It is reported p21 inhibits the kinase activity of CDK2–cyclin A and CDK1–cyclin A, which is essential for progression of S phase as well as to enter G2 phase. p27 also involved in the cell cycle arrest is highly expressed in these cells. Forkhead box O-3a (FOXO3a) transcription factor which mediates apoptosis, inhibits the proliferation and also the expression of cell cycle regulatory proteins showed high expression in SHED cells incubated with low glucose. Our results suggests that nutrient deprivation leads to the cell cycle arrest in S-phase via high expression of cyclin inhibitors, low expression of cyclin along with the elevated expression of transcription factor FOXO3. FOXO3 expression is directly regulated by the nutrient sensor AMP-activated protein kinase (AMPK) [27]. So, we further aimed to explore the involvement of AMPK in nutrient deprived SHEDs.

AMP-activated protein kinase (AMPK) plays an important role in maintaining homeostasis of energy, controls the metabolic pathways and also nutrient supply. AMPK is often known as cellular energy sensor as it can be activated by various conditions such as decrease in cellular energy levels, low glucose level, hypoxia and exposure to toxins [28]. AMPK primarily controls the balance of ATP and inhibits the anabolic pathways [29]. AMPK also regulates transcription of several metabolomic kinases. Recent studies showed the involvement of AMPK in cell polarity and cytoskeletal dynamics [30]. As AMPK plays major roles in cell growth regulation as well as metabolic reprograming, there have been several studies ongoing to determine the therapeutic potential of AMPK. In our study we further aimed to investigate the expression of AMPK in nutrient deprived SHEDs. We found higher activity of AMPK in these cells, previously it has been reported the AMPK mediated VEGF expression as well as angiogenesis [31]. However, there was no significant difference in expression of VEGFA between the two conditions. Dorsomorphin, a small molecule, has been widely employed as a selective AMPK inhibitor. Dorsomorphin was utilized to suppress AMPK phosphorylation in response to low glucose levels in the blood. After pretreatment with dorsomorphin, p-AMPK was down-regulated, as shown in the results. Low glucose serum, on the other hand, did not prevent dorsomorphin from downregulating p-AMPK. These findings suggested that a low glucose-serum state activates the AMPK pathway, as seen by the production of p-AMPK even after dorsomorphin inhibition. AMPK also regulates the expression of LIF, which is involved in maintaining embryonic stem cell renewal [32,33]. Activation of AMPK might be playing a role in the differential proliferative, differentiation potential of SHEDs in serum deprived medium. Further experiments have to be carried out to understand the underlying mechanisms.

Further we examined the expression of stemness markers in nutrient deprived SHEDs, we found increased expression of c-Myc and Sox2. However, there was no significant difference between the expression of Oct4 and Nanog. Our results imply there is an increase stemness properties in nutrient deprived SHEDs. Park et al., reported the key role of Sox2 in determining lineage determination in mesenchymal cells [34]. Runx2, the most important osteogenic transcriptional factor, and is a key determinant in the early osteogenic differentiation is an important marker of early osteogenic differentiation [35]. We observed high expression of Sox2, low expression of Runx2 and STRO1 suggesting serum and glucose deprivation enhanced the chondrogenic differentiation potential but reduced the osteogenic differentiation potential of SHEDs. Our study showed that nutrient deprivation of SHEDs led to reduced proliferation, increased apoptosis, activation of stress sensing molecule AMPK and induced distinct differentiation lineages in SHEDs.

## 5. Conclusions

SHEDs are mesenchymal cells derived from the exfoliated human deciduous teeth pulp and are widely under investigation for complementing the traditional MSC sources. Many microenvironmental factors such as ischemic shock, hypoxia and low glucose level influence the homing of MSCs in transplanted system. In this study, we investigated the effects of serum deprivation and low glucose in determining the fate of SHEDs. Our results suggested, the nutrient deprivation and low glucose level affected the proliferation, apoptosis, cell cycle and differentiation potency of SHEDs. Our result is a preliminary study showing the effects of low serum and glucose in in-vitro models. Our results suggest low nutrient and low glucose affect the fate of transplanted MSCs in host cell microenvironment. However, further in-vivo studies have to be carried out to understand the multifactorial effects of microenvironment on SHEDs.

## Figures and Tables

**Table 1:** List of primers.

Gene	Forward Primer	Reverse Primer
CCNB1	5′-GAC CTG TGT CAG GCT TTC TCT G-3′	5′-GGT ATT TTG GTC TGA CTG CTT GC-3′
CCNE1	5′-TGT GTC CTG GAT GTT GAC TGC C-3′	5′-CTC TAT GTC GCA CCA CTG ATA CC-3′
CCND1	5′-TCT ACA CCG ACA ACT CCA TCC G-3′	5′-TCT GGC ATT TTG GAG AGG AAG TG-3′
CCNA2	5′-CTC TAC ACA GTC ACG GGA CAA AG-3′	5′-CTG TGG TGC TTT GAG GTA GGT C-3′
CDKN1A	5′-AGG TGG ACC TGG AGA CTC TCA G-3′	5′-TCC TCT TGG AGA AGA TCA GCC G-3′
CDKN1B	5′-ATA AGG AAG CGA CCT GCA ACC G-3′	5′-TTC TTG GGC GTC TGC TCC ACA G-3′
FOXO1	5′-CTA CGA GTG GAT GGT CAA GAG C-3′	5′-CCA GTT CCT TCA TTC TGC ACA CG-3′
FOXO3	5′-TCT ACG AGT GGA TGG TGC GTT G-3′	5′-CTC TTG CCA GTT CCC TCA TTC TG-3′
VEGFA	5′-TTG CCT TGC TGC TCT ACC TCC A-3′	5′-GAT GGC AGT AGC TGC GCT GAT A-3′
LIF	5′-AGA TCA GGA GCC AAC TGG CAC A-3′	5′-GCC ACA TAG CTT GTC CAG GTT G-3′
IL8	5′-GTG CAG TTT TGC CAA GGA GT-3′	5′-TTA TGA ATT CTC AGC CCT CTT CAA-3′
IL10	5′-TCT CCG AGA TGC CTT CAG CAG A-3′	5′-TCA GAC AAG GCT TGG CAA CCC A-3′
PTCH1	5′-GCT GCA CTA CTT CAG AGA CTG G-3′	5′-CAC CAG GAG TTT GTA GGC AAG G-3′
FZD1	5′-GCT TTG TGT CGC TCT TCC GCA T-3′	5′-TAC AGC ACG CTG AAG ACG CCA A-3′
OCT3/4	5′-CCT GAA GCA GAA GAG GAT CAC C-3′	5′-AAA GCG GCA GAT GGT CGT TTG G-3′
SOX2	5′-GCT ACA GCA TGA TGC AGG ACC A-3′	5′-TCT GCG AGC TGG TCA TGG AGT T-3′
NANOG	5′-CTC CAA CAT CCT GAA CCT CAG C-3′	5′-CGT CAC ACC ATT GCT ATT CTT CG-3′
MYC	5′-CCT GGT GCT CCA TGA GGA GAC-3′	5′-CA GAC TCT GAC CTT TTG CCA GG-3′
SOX9	5′-GCC GAA AGC GGG CTC GAA AC-3′	5′-AAA AGT GGG GGC GCT TGC ACC-3′
RUNX2	5′-GTG CCT AGG CGC ATT TCA-3′	5′-GCT CTT CTT ACT GAG AGT GGA AGG-3′
PPARG	5′-AGC CTG CGA AAG CCT TTT GGT G-3′	5′-GGC TTC ACA TTC AGC AAA CCT GG-3′
ACTB	5′-AGA GCT ACG AGC TGC CTG AC-3′	5′-AGC ACT GTG TTG GCG TAC AG-3′
